# Trends in Incidence of Hip Fracture and Hip Replacement in Denmark, 1996 to 2018

**DOI:** 10.1001/jamanetworkopen.2024.9186

**Published:** 2024-05-01

**Authors:** Troels Mygind Jensen, Jacob Krabbe Pedersen, Frans Boch Waldorff, Jens Søndergaard, Søren Overgaard, Kaare Christensen

**Affiliations:** 1 Research Unit for General Practice, Department of Public Health, University of Southern Denmark, Odense; 2 Danish Aging Research Center, Department of Public Health, University of Southern Denmark, Odense; 3 Research Unit for Epidemiology, Biostatistics and Biodemography, Department of Public Health, University of Southern Denmark, Odense; 4 Research Unit for General Practice and Section of General Practice, Department of Public Health, University of Copenhagen, Copenhagen, Denmark; 5 Department of Orthopaedic Surgery and Traumatology, Copenhagen University Hospital, Bispebjerg, Denmark; 6 Department of Clinical Medicine, Faculty of Health and Medical Sciences, University of Copenhagen, Copenhagen, Denmark

## Abstract

**Importance:**

The past several decades have witnessed substantial changes in treatments that are particularly relevant for older patients.

**Objectives:**

To assess changes in national-level incidence rates of fracture- and musculoskeletal-related (ie, arthritis-related) hip replacement procedures for individuals aged 40 to 104 years over a 23-year period in Denmark.

**Design, Setting, and Participants:**

This cohort study used national Danish health registers to include the Danish population aged 40 to 104 years from January 1, 1996, to December 31, 2018. Data were analyzed from May 31, 2022, to February 14, 2024.

**Main Outcomes and Measures:**

Age- and period-specific incidence rates of hip fracture and hip replacement stratified on fracture-related vs arthritis-related indication.

**Results:**

From 1996 to 2018, a total of 3 664 979 individuals were followed up for a mean (SD) of 14.6 (7.7) years, resulting in a follow-up time of 53 517 861 person-years and 158 982 (first) hip fractures, of which 42 825 involved fracture-related hip replacement procedures. A further 104 422 individuals underwent arthritis-related hip replacement. During the first 2 decades of the 21st century, hip fracture rates declined by 35% to 40% for individuals aged 70 to 104 years, and the proportion of the population undergoing fracture-related hip replacement increased by 50% to 70%, with modest variation across those aged 75 to 99 years. Rates of arthritis-related hip replacements peaked for individuals aged 75 to 79 years, but with the largest relative rate increase (75%-100%) occurring for those aged 80 to 94 years, primarily from 2001 to 2015, whereafter it remained nearly unchanged. The decline in rates of arthritis-related hip replacement after 75 to 79 years of age was gradual and did not suggest an upper age limit for access to arthritis-related hip replacement.

**Conclusions and Relevance:**

The findings of this cohort study suggest that during the past several decades in Denmark, the incidence of hip fractures declined by 35% to 40% among patients aged 80 to 104 years, while the proportion receiving fracture-related hip replacement remained relatively constant after 75 years of age. During the first decades of the 21st century, arthritis-related hip replacement incidence increased by 50% to 100% among older patients and stabilized hereafter, with no apparent cutoff age for this type of procedure. These patterns indicate a positive overall trend with declining hip fracture incidence over the last decades in Denmark, and the observed hip replacement incidence suggests that age is currently not a major determining factor guiding this type of surgery.

## Key Points

**Question:**

How has incidence of hip fracture and hip replacement developed over the past several decades in Denmark?

**Findings:**

In this cohort study including 3 664 979 individuals aged 40 to 104 years, hip fracture incidence rates among patients aged 80 to 104 years declined by 35% to 40% during the decades since 1996 in Denmark, while the proportion undergoing fracture-related hip replacement increased by 50% to 70% and with moderate age dependence after 75 years of age. For arthritis-related hip replacement, rates increased by 60% to 100% among individuals aged 75 to 99 years.

**Meaning:**

These findings suggest that hip fracture incidence has declined in Denmark since 1996, and the observed hip replacement incidence suggests that age is not a major determining factor guiding this type of surgery.

## Teaser

This registry-based cohort study examines trends in rates of hip fracture and hip replacement surgery among adults in Denmark 40 years and older since 1996.

## Introduction

Given the rapid aging of populations globally, it has become increasingly important to ensure access to health care services throughout the life course^1^ to ensure equal access to treatment. Health inequities pertaining to age are, however, ubiquitous and have been extensively documented.^2,3,4,5,6,7^

A recent study^8^ established that even among patients older than 90 years, elective total hip replacement (due to osteoarthritis) may be appropriately considered, if patients are carefully selected. Another recent study^9^ recommended that, despite higher mortality compared with younger groups, nonagenarian patients should not be denied total hip replacement. This recommendation was based on patient satisfaction and pain relief being the same or better among this age group.^9^

For patients with hip fracture in Denmark, surgery is most often performed.^10^ However, on an international scale, the rates of nonoperative treatment of hip fracture vary significantly, from 1% to 25%.^11,12^ The decision to use nonoperative management for hip fracture, particularly among patients aged 80 to 104 years, may be highly challenging due to ethical, cultural, and legal issues,^13,14^ and localization of the fracture may determine the preferred type of surgery.^15,16^ These concerns should be considered within the context of hip fracture management, as hip fractures rank among the top 10 causes of disability globally, currently affecting 4.5 million people and are anticipated to increase to 21 million individuals within the next few decades.^17^

In Denmark, studies have observed increased treatment activity in the older patients (eg, increased use of prescription medication during the last years of life)^18^ as well as increases in hospitalizations and surgical procedures.^19^ Therefore, with a particular focus on patients aged 80 to 104 years, we examined both rates of hip fracture and rates of hip replacement surgery, a type of surgical procedure that is also indicated for older patients and largely based on either fracture-related or musculoskeletal-related indications. Hip replacement related to fractures is most commonly associated with falls, whereas musculoskeletal-related hip replacement is performed principally due to osteoarthritis (hereinafter referred to as arthritis-related).

## Methods

This cohort study was approved by Statistics Denmark and by the internal review board of the University of Southern Denmark, SDU Research and Innovation Organisation, which waived the need for informed consent owing to the use of deidentified registry data. The study followed the Strengthening the Reporting of Observational Studies in Epidemiology (STROBE) reporting guideline.

### Study Population

Data were obtained from the Danish universal health care system, which has had residency-based entitlement since the early 1970s^20^ and provides prospectively collected, detailed information on health services . The study population was based on the entire Danish-born population 40 years and older and living in Denmark on or after January 1, 1996. Data on race and ethnicity were not available from Statistics Denmark. With the study of both fracture and hip replacement rates, only individuals with no previous events (either hip fracture or hip replacement) were included. Information on hip fractures and surgical procedures (hemiarthroplasties and total hip arthroplasties, hereinafter referred to as hip replacement) were obtained via Statistics Denmark through linkage to the Danish Civil Registration System^21^ (which stores personal information on all Danish citizens through a unique personal identification number, enabling unambiguous linkage at an individual level), and to the Danish National Patient Registry^22^ (which contains information on all inpatients in Danish hospitals throughout the period from 1977 to December 31, 2018, thus allowing detection of hip fractures and hip replacements in the 19 years prior to 1996). From 1977 to 1993, diseases in the Danish National Patient Registry were classified according to the *International Classification of Diseases, Eighth Revision* (*ICD-8*), and from 1994 onward according to the *International Statistical Classification of Diseases, Tenth Revision* (*ICD-10*). From 1977 to 1995, surgical procedures were coded according to the 3 consecutive editions of the Danish Classification of Surgical Procedures and Therapies (DCSPT). Since 1996, surgical procedures have been coded according to the Danish version of the Nordic Medico-Statistical Committee Classification of Surgical Procedures (NOMESCO).^23^

### Outcomes

For incidence of hip fracture and hip replacement, only the patient’s first-occurring event was considered, so that this fracture formed the basis for the censoring of all subsequent fractures and hip replacements. Events occurring before 40 years of age were also censored. If a hip fracture and a hip replacement occurred simultaneously, both events counted in the calculations of fracture and hip replacement rates.

Fracture was identified as the first hospitalization for hip fracture as the primary diagnosis (*ICD-10* codes S72.0, S72.1, and S72.2; *ICD-8* code 820) (Table) where the fracture was simultaneously associated with a relevant surgical procedure (NOMESCO operation codes NFB, NFG09, and NFJ; DCSPT operation codes 730.20, 732.00, 734.00, 736.00, 736.20, 739.00, 739.20, 739.40, 749.40, 826.80, 827.00, and 827.40). The surgical treatments were identified by linking records of each verified fracture to all surgical procedures recorded during the period of hospitalization. For these verified fractures, the proportions for which surgical procedures included osteosynthesis of hip fracture (NOMESCO operation codes NFJxy for x = 2-9 and y = 0-3) or hip replacement (NOMESCO operation codes NFB; DCSPT operation codes 700.30-40 and 700.80-89) were calculated. To obtain a simplified definition of fracture, a sensitivity analysis of the fracture rates and proportions was conducted requiring only a primary diagnosis of fracture with no inclusion of subsequent treatment.

**Table.** Coding for Hip Fractures and Surgical Procedures

Event by source of code	Code
Hip fractures	
*ICD-8* (1977-1993)	820
*ICD-10* (1994 onward)	S72.0, S72.1, S72.2
Procedural codes for verifying hip fracture^a^	
DCSPT (1977-1995)	700.30-39, 700.40, 700.80-89, 736.00, 736.20, 756.36-38, 749.40, 826.80, 827.00, 827.40
NOMESCO (1996 onward)	NFB, NFG09, NFJ
Osteosynthesis of hip fracture	
DCSPT (1977-1995)	Not relevant
NOMESCO (1996 onward)	NFJ2y, NFJ3y, NFJ4y, NFJ5y, NFJ6y, NFJ7y, NFJ8y, NFJ9y (y = 0-3)
Hip replacement	
DCSPT (1977-1995)	700.30-39, 700.40, 700.80-89
NOMESCO (1996 onward)	NFB

Abbreviations: DCSPT, Danish Classification of Surgical Procedures and Therapies; *ICD-8*, *International Classification of Diseases, Eighth Revision*; *ICD-10*, *International Statistical Classification of Diseases, Tenth Revision*; NOMESCO, Danish version of the Nordic Medico-Statistical Committee Classification of Surgical Procedures.

^a^ Verification of hip fracture required a hip fracture diagnosis in combination with a fracture-related surgical procedure.

Incidence rates for fracture-related hip replacement were estimated using only the first hip replacement of each individual and restricting to hospitalization where hip fracture was the primary diagnosis. In addition to rates of fracture-related hip replacement, rates of arthritis-related hip replacement were estimated. Arthritis-related hip replacement was defined by a hip replacement associated with a primary diagnosis of a musculoskeletal disease (*ICD-10* code M00-99). Osteoarthritis of hip (*ICD-10* code M16) comprised 97% of these diagnoses.

Individuals were censored at time of event (hip fracture, hip replacement, or both), death, emigration, or the end of study period, whichever came first. All incidence rates and proportions were calculated for 5-year intervals, from 40 to 44 years of age until 100 to 104 years of age, and 5-calendar-year intervals, from 1996 to 2000 until 2016 to 2018 (most recent interval spans 3 years due to data availability).

### Statistical Analysis

Analyses for the present study were performed from May 31, 2022, to February 14, 2024, using Stata, version 18.0 (StataCorp LLC). Because the study was based on nationwide register data with virtually complete coverage, considerations regarding statistical power were mostly not necessary.

## Results

From 1996 to 2018, a total of 3 664 979 individuals (51.1% female and 48.9% male) were followed up for a mean of 14.6 (7.7) years, resulting in 158 982 (first) hip fractures for which 42 825 (26.9%) fracture-related hip replacement procedures were performed. Descriptives of follow-up time are shown in the eTable in Supplement 1. For a further 104 422 individuals (65.7%), hip replacements were related to musculoskeletal disease (arthritis-related).

**Figure 1.**
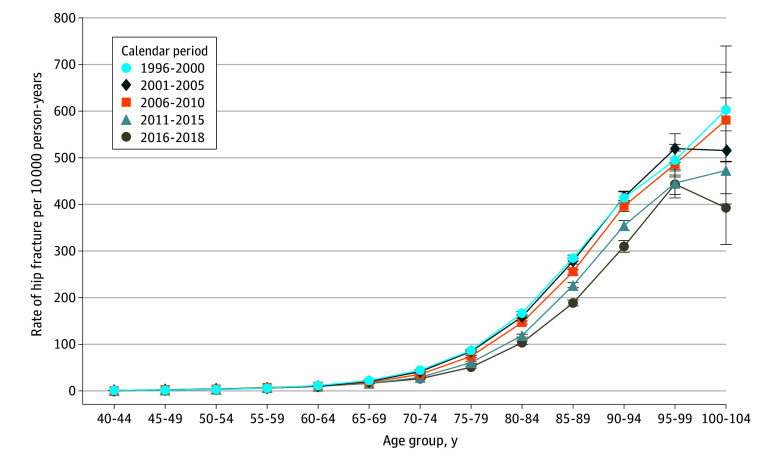
Age-Specific Hip Fracture Incidence Rates per 10 000 Person-Years in Denmark Among Patients Aged 40 to 104 Years From 1996 to 2018 Rates were calculated using only the first fracture of each individual in the study population. The sole purpose of lines connecting point estimates is to ease the comparison of pattern of rates between different calendar periods, while the lines do not represent estimates rates other than at the point estimates. Error bars indicate 95% CIs.

The incidence rates for hip fracture increased with age across the 5 calendar periods (Figure 1). Incidence decreased across the periods, resulting in substantially lower rates in the most recent period (2016-2018). For example, for patients aged 80 to 84 years, the decline in hip fracture incidence rates from the 1996-2000 to 2016-2018 periods corresponded to a 38% relative reduction.

**Figure 2.**
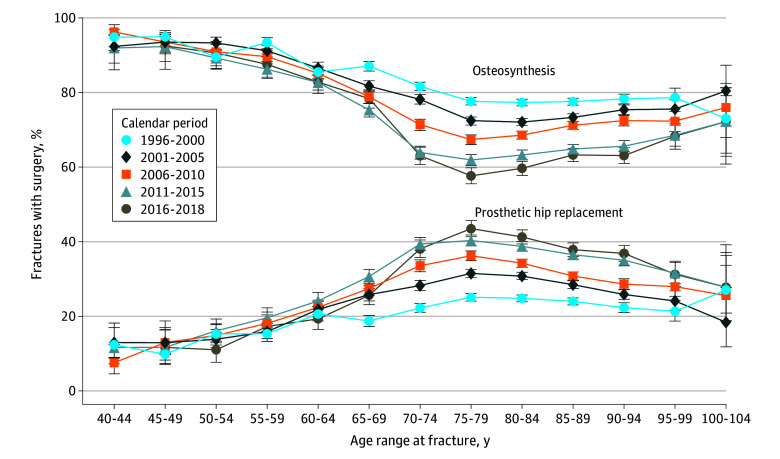
Proportions of Hip Fractures Treated With Osteosynthesis and Total Hip Replacement in Denmark Among Patients Aged 40 to 104 Years From 1996 to 2018 Only the first fracture of each individual in the study population was included. The sole purpose of lines connecting point estimates is to ease the comparison of pattern of rates between different calendar periods, while the lines do not represent estimates rates other than at the point estimates. Error bars indicate 95% CIs.

The proportion of patients undergoing hip replacement following a hip fracture increased by 50% to 70% from 1996 to 2018. For example, for patients aged 80 to 84 years, the proportion of hip replacement based on hip fracture increased from 25% in 1996 to 2000 to 41% in 2016 to 2018 (Figure 2). The moderately horizontal curves during all periods for patients aged 75 to 104 years show that receiving hip replacement after hip fracture is not markedly dependent on age.

In the sensitivity analysis, where fracture was defined less restrictively, rates were slightly higher for all ages and periods, but the interpretation of the rates was similar—that is, increasing rates with age and decreasing rates in more recent years. Similarly, for the proportion of fractures followed by hip replacement or osteosynthesis, even if only about 90% of patients with fractures underwent 1 of the 2 procedures, the percentage was not markedly dependent on age and period. Therefore, the corresponding curves in Figure 2 were slightly lower in the sensitivity analysis but still not strongly dependent on age after 75 years (in contrast to the dependence between age and arthritis-related hip replacement).

**Figure 3.**
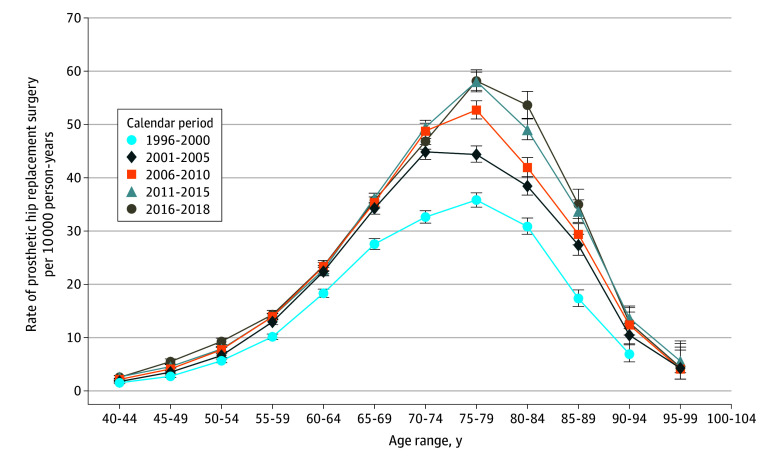
Rates of Arthritis-Related Hip Replacement per 10 000 Person-Years in Denmark Among Patients Aged 40 to 104 Years From 1996 to 2018 Arthritis-related hip replacement was defined as a patient’s first hip replacement accompanied by a primary diagnosis of a musculoskeletal disease (*International Statistical Classification of Diseases, Tenth Revision,* code M00-99). The sole purpose of lines connecting point estimates is to ease the comparison of pattern of rates between different calendar periods, while the lines do not represent estimates rates other than at the point estimates. Error bars indicate 95% CIs.

The arthritis-related total hip replacement procedure rates increased for all age groups during the last decades of the 20th century, peaking at ages 75 to 79 years in all periods and with the relatively largest increases (75%-100%) among patients aged 80 to 94 years (Figure 3). For example, from the 1996-2000 period to the 2016-2018 period, for patients aged 90 to 94 years, the increase in rates of arthritis-related hip replacement procedures (from 6.4 to 12.8 per 10 000 person-years) corresponded to an 84% increase. (The estimates and 95% CIs shown in Figures 1 to 3 are given without connecting lines in eFigures 1 to 3 in Supplement 1).

**Figure 4.**
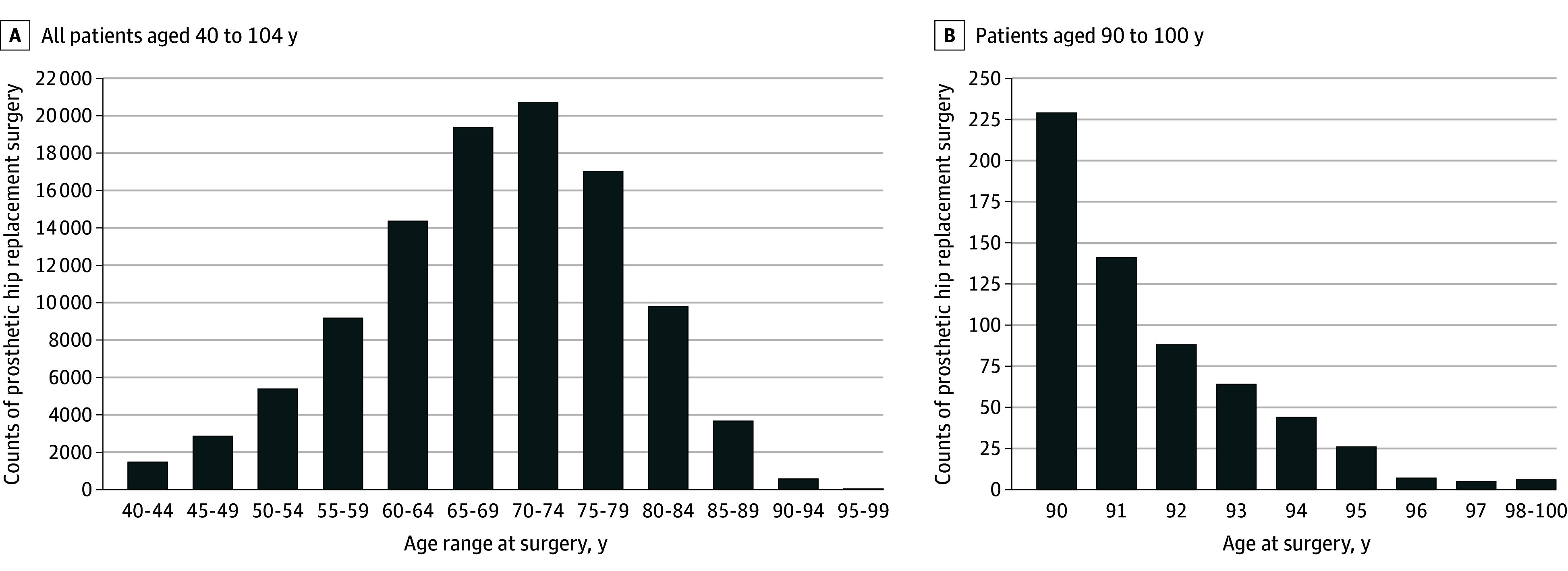
Counts of Hip Replacement Related to Arthritis in Denmark Among Patients Aged 40 to 104 Years From 1996 to 2018 A, Counts among patients aged 40 to 99 years in 5-year age intervals. B, Counts among patients 90 years or older in 1-year age intervals (except last interval for ≥98 years). Arthritis-related hip replacement was defined as a patient’s first hip replacement accompanied by a primary diagnosis of a musculoskeletal disease (*International Statistical Classification of Diseases, Tenth Revision*, code M00-99).

Additionally, the decrease in arthritis-related hip replacement rates after 75 to 79 years of age was gradual, with the absence of a sharp decline, even when focusing on those 90 years or older (Figure 4). This finding suggests that a specific 2-digit age cutoff has not existed for this procedure.

## Discussion

In the first decades of the 21st century in Denmark, arthritis-related hip replacement incidence almost doubled among older patients, with the most pronounced relative increases at the oldest ages. Furthermore, the decline in rates after 75 to 79 years of age was gradual, with no indication of a 2-digit cutoff age for hip replacement related to arthritis. During the first 2 decades of the 21st century, the incidence of hip fractures declined while the proportion receiving hip replacement after fracture increased with only mild age dependence after 75 years of age.

In our study, we observed a substantial decline in the rates of hip fractures in Denmark. The decline is of particular significance from a Danish public health perspective, as a 2012 study observed that Denmark had the highest hip fracture incidence rates among 63 countries investigated.^24^

A Danish study^25^ examining hip fracture incidence rates from 1997 to 2006 among individuals 60 years or older reported an average decrease of 20% and 22% in men and women, respectively; however, our study covers an expanded period, which could explain the more substantial decrease. The observed decline may represent the accumulated result of several factors, including smoking cessation, fall prevention strategies, and improved bone health due to antiosteoporosis treatment (eg, bisphosphonates), which entered Danish clinical practice in the mid-1990s.^26^ However, regarding the latter, it should be noted that a Danish study^27^ observing a 30% decline in hip fracture rates during 2005 to 2015 found that 80% of the decline was likely attributable to factors other than pharmacological intervention; rather, the authors suggest that the hip fracture rate decline may be due to a short-term decline in population risk level. Similarly, a recent Norwegian study^28^ found that one-fifth of the observed hip fracture rate decline from 1999 to 2019 was due to antiosteoporosis medication, and the study primarily attributed the decline to reductions in major risk factors, including increased population body mass index, increased prevalence of persons living with total hip prostheses, increased proportion of physically active individuals, as well as a decline in smoking prevalence. Importantly, the observed decline is likely to have a significant effect on health economics, reducing the substantial care costs associated with hip fracture.^29^

We observed an increase in the proportion of patients receiving fracture-related hip replacement. Interestingly, we also found that for the proportion receiving fracture-related hip replacement, there was no sharp decline in the age group aged 70 to 99 years throughout each period, suggesting that there is no fixed age limit to fracture-related hip replacement in any of the periods studied and that there is no age at which the rate suddenly drops to 0.

For arthritis-related hip replacement, the pattern was related to age, with rates peaking at 75 to 79 years of age and decreasing at the oldest ages in all periods. However, the largest relative increase was observed for 85 to 99 years of age, indicating a shift toward more intense treatment of this patient group. Although the rates for surgery were lower among patients aged 80 to 104 years, age may not be decisive in determining access to treatment. As for all invasive procedures, these interventions carry inherent benefits and risks, and the decision to treat (especially in cases of concurrent disability, frailty, or comorbidity) is often the result of a balance of clinical judgements, including patients’ preferences and decisions on whether to opt out of treatment.^30^ Surgery related to osteoarthritis may be complex and shaped by different factors. Plausible explanations for the increased surgical activity may reflect changing attitudes from society and among older patients and in part may reflect changed treatment practices, possibly due to technological advances (eg, improved anesthesiologic and surgical procedures),^31,32,33,34,35,36^ allowing for treatment of individuals whose frailty previously precluded them from having the procedure. Also, the demographic composition and the general improvement in health and functioning among the oldest people in Denmark is likely to have contributed to the development.^37,38^ Additionally, for both types of hip replacements, the lower incidence of first hip replacement among individuals aged 80 to 104 years may at least in part be due to the increased rate of hip replacement at younger ages, indicating that the proportion of individuals who have already received a hip replacement before reaching an advanced age has increased.

A Danish study^26^ reported slight increases in hip fracture rates among individuals older than 70 years from 1977 to 1995, with a subsequent stagnation in rates from 1955 to 1999. Another Danish study^39^ reported a decrease in the incidence rate of first hip fracture (2.4% per year for men and 1.8% per year for women) from 1996 to 2003, with the greatest decrease of 3.4% per year observed for women aged 80 to 84 years.

The substantial decline in hip fracture rates during the 21st century observed in our study can be viewed as an extension of those previous findings, showing that the previous rate increases have been reversed. Indeed, these breaks in increasing trends have been mirrored across Scandinavia^39,40,41^ and the Western world^42^ during the last 1 or 2 decades. A study investigating age, period, and cohort effects across Denmark from 1980 to 2010 and across Sweden from 1987 to 2011 found decreasing or stable age-standardized rates of hip fractures.^43^ Also, the authors suggested that an increase in hip fracture rates should be expected owing to higher relative risks among the more recently born cohorts investigated in their study. However, the findings in our study indicate that the opposite has occurred in Denmark, with a decrease in hip fracture rates for the subsequent period, covering the late 2000s through 2018.

Two reports (from 2012 and 2014) by the Royal College of Surgeons^44,45^ explored variations in access to surgery among older people. The first report, from 2012,^44^ observed a marked decrease from 2008 to 2011 in the rates of arthritis-related surgical procedures for patients older than 65 years for a range of conditions, including cancer and heart disease, and a consistent decrease over the 4 years examined in rates of hip replacement surgery for patients in their late 70s and older.

Interestingly, the 2014 report^45^ found that during 2011 to 2012, the rate of hip replacement procedures was higher for individuals 75 years or older than for those aged 65 to 74 years, although the authors point out that data include both fracture-related and arthritis-related procedures, and therefore the overall rise may have been due to an increase in fracture-related hip replacement among those 75 years or older. In our study, we observed a peak in the rates of non–fracture-related procedures at 75 to 79 years of age, although a substantial relative increase was observed among patients aged 80-99 years in the first 2 decades of the 21st century. For fracture-related procedures, our results mirror the findings of the UK study in that they show a higher proportion of patients 75 years or older undergoing surgery compared with patients aged 65 to 74 years, which is to be expected given that fractures among patients in the latter age group have been treated with osteosynthesis rather than hip replacement. The novel finding in our study is that the proportion of patients undergoing hip replacement after a hip fracture does not exhibit a sharp decline among individuals aged 75 to 104 years. This, in combination with the marked increase in arthritis-related hip replacement among older people and no indication of a fixed age limit, suggests that chronological age is not a major determining factor guiding the clinical decision to perform hip replacement.

### Strengths and Limitations

Our findings are strengthened by the use of high-quality data from nationwide registers, providing excellent coverage with virtually no loss to follow-up, thus reducing the risk of selection bias. When interpreting our results, some limitations also should be considered. The Danish National Patient Registry contains only data collected during hospital admissions for hip fractures and replacement. Data on other conditions that may have provided a more detailed view of clinical decision-making, such as comorbidities, disability, and frailty, were not included in our study. As the study was conducted in the context of a public tax-financed health care system, the applicability of our results in countries with nonuniversal health care is unclear.

## Conclusions

The findings of this cohort study suggest that age did not solely determine access to treatment; however, the observed increased treatment intensity among older patients over the past several decades carries a dual perspective. This development may entail detrimental consequences, including overdiagnosis and overtreatment, but on the other hand may contribute to ensuring equal access to treatment. Also, our findings warrant further examinations of other types of surgical procedures relevant to older patients, such as knee replacement, cataract surgery, and coronary bypass, to gain a more comprehensive view of ageism in treatment. From a public health standpoint, our study provides a view of the access to a potentially life-enhancing surgical procedure, contributing to the maintenance of independence among older patients and helping them to live longer with better functioning.

## Notes

Supplement 1. **eTable.** Descriptives of the Follow-Up Time Distribution (in Years) **eFigure 1.** Age-Specific Hip Fracture Incidence Rates (per 10 000 Person-Years) **eFigure 2.** Proportions of Hip Fractures for Which Treatment Included Hip Replacement or Osteosynthesis **eFigure 3.** Rates of Arthritis-Related Hip Replacement (per 10 000 Person-Years)

Supplement 2.
Data Sharing Statement
