# Supplementary material for: Trends in Incidence of Hip Fracture and Hip Replacement in Denmark, 1996 to 2018
Source: JAMA Netw Open. 2024 May 1;7(5):e249186. doi: 10.1001/jamanetworkopen.2024.9186 (PMC11063804; doi:10.1001/jamanetworkopen.2024.9186)
Supplement: Supplement 1. — eTable. Descriptives of the Follow-Up Time Distribution (in Years) eFigure 1. Age-Specific Hip Fracture Incidence Rates (per 10 000 Person-Years) eFigure 2. Proportions of Hip Fractures for Which Treatment Included Hip Replacement or Osteosynthesis eFigure 3. Rates of Arthritis-Related Hip Replacement (per 10 000 Person-Years) [file jamanetwopen-e249186-s001.pdf]

Supplementary Online Content

Jensen TM, Pedersen JK, Waldorff FD, Søndergaard J, Overgaard S, Christensen K. Trends in incidence of hip fracture and hip replacement in Denmark, 1996 to 2018. *JAMA Netw Open*. 2024;7(5):e249186. doi:10.1001/jamanetworkopen.2024.9186

eTable. Descriptives of the Follow-Up Time Distribution (in Years)

eFigure 1. Age-Specific Hip Fracture Incidence Rates (per 10 000 Person-Years)

eFigure 2. Proportions of Hip Fractures for Which Treatment Included Hip Replacement or Osteosynthesis

eFigure 3. Rates of Arthritis-Related Hip Replacement (per 10 000 Person-Years)

This supplementary material has been provided by the authors to give readers additional information about their work.

© 2024 Jensen TM et al. *JAMA Network Open*.

eTable. Descriptives of the Follow-Up Time Distribution (in Years)

		Follow-up-time					Age at study start					Age at end of study				
	**Individuals**	**Mean**	**Median**	**Q1**	**-**	**Q3**	**Mean**	**Median**	**Q1**	**-**	**Q3**	**Mean**	**Median**	**Q1**	**-**	**Q3**
**Men**	1.790.950	14,4	15,2	7,6	-	23,0	50,8	45,4	40,0	-	58,9	65,2	65,3	53,5	-	76,1
**Women**	1.874.029	14,8	15,9	8,0	-	23,0	52,9	47,8	40,0	-	63,3	67,7	68,2	55,2	-	79,5
**All**	3.664.979	14,6	15,6	7,8	-	23,0	46,6	51,9	40,0		61,1	66,5	66,7	55,4		77,8

Note: 28.4% of the study population were under risk in the entire study period from 1996 to 2018 (23 years), while the follow-up follow-up-time of the remaining population was uniformly distributed on the interval 0 to 23 years. For each sex, the same observation held, with the slight difference that 27.2% of men and 29.7% of women were under risk in the entire study period.

© 2024 Jensen TM et al. *JAMA Network Open*.

eFigure 1. Age-Specific Hip Fracture Incidence Rates (per 10 000 Person-Years)

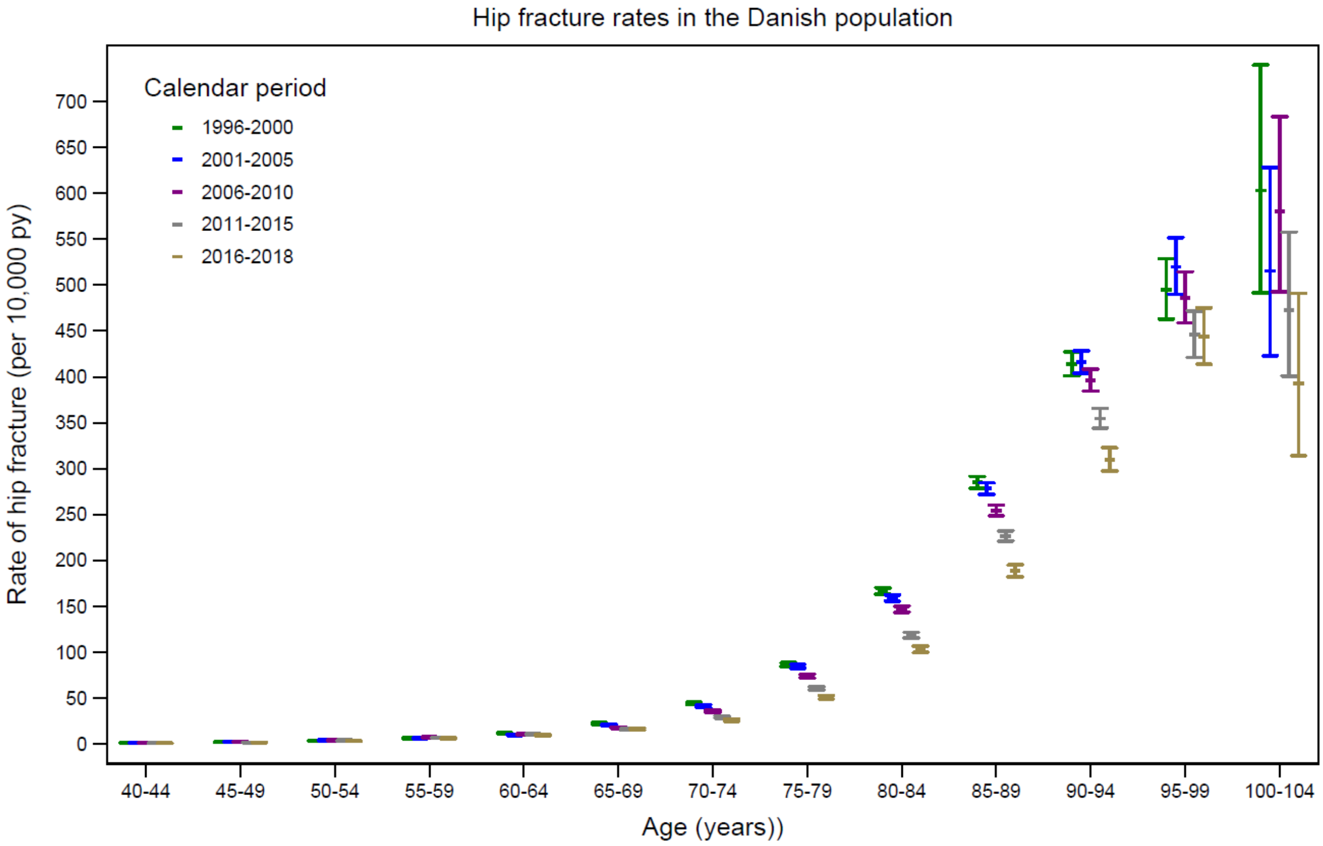

© 2024 Jensen TM et al. *JAMA Network Open*.

eFigure 2. Proportions of Hip Fractures for Which Treatment Included Hip Replacement or Osteosynthesis

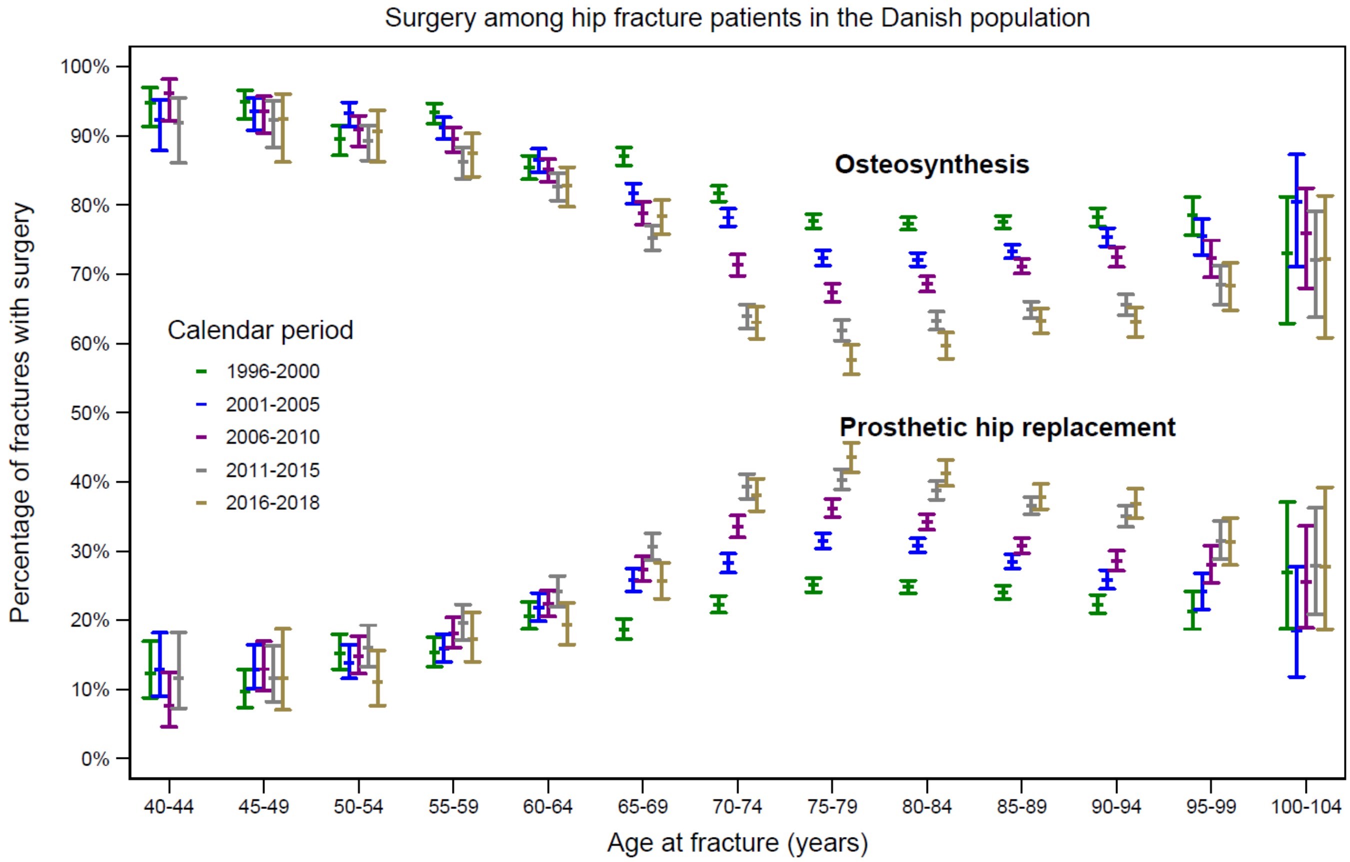

© 2024 Jensen TM et al. *JAMA Network Open*.

eFigure 3. Rates of Arthritis-Related Hip Replacement (per 10 000 Person-Years)

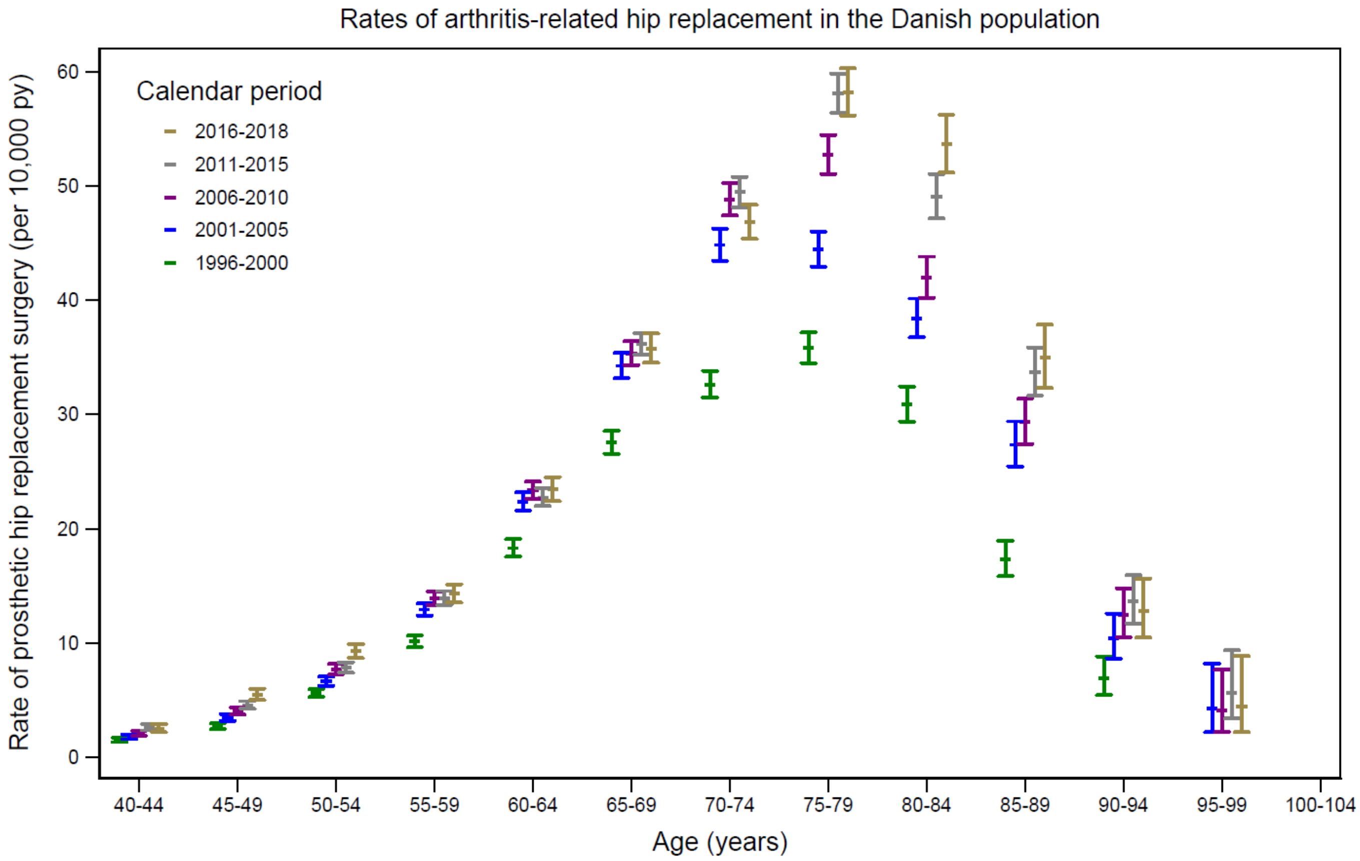

© 2024 Jensen TM et al. *JAMA Network Open*.
