# Supplementary material for: Trends in Incidence of Hip Fracture and Hip Replacement in Denmark, 1996 to 2018
Source: JAMA Netw Open. 2024 May 1;7(5):e249186. doi: 10.1001/jamanetworkopen.2024.9186 (PMC11063804; doi:10.1001/jamanetworkopen.2024.9186)
Supplement: Supplement 2. — Data Sharing Statement [file jamanetwopen-e249186-s002.pdf]

Data Sharing Statement

Jensen. Trends in Incidence of Hip Fracture and Hip Replacement in Denmark, 1996 to 2018. *JAMA Netw Open*. Published May 01, 2024. doi:10.1001/jamanetworkopen.2024.9186

Data
Data available: No
